# Comparative Evaluation of Shear Bond Strength of Nanohybrid Composite Restoration After the Placement of Flowable Compomer and Composite Using the Snowplow Technique

**DOI:** 10.7759/cureus.28663

**Published:** 2022-09-01

**Authors:** Meghna Dugar, Anuja Ikhar, Pradnya Nikhade, Manoj Chandak, Nidhi Motwani

**Affiliations:** 1 Department of Conservative Dentistry and Endodontics, Sharad Pawar Dental College and Hospital, Datta Meghe Institute of Medical Sciences, Wardha, IND

**Keywords:** snowplow technique, shear bond strength, nanohybrid composite, flowable compomer, flowable composite

## Abstract

Aim: Based on the importance of bonding during restoration, this in vitro study aimed to evaluate the difference in shear bond strength of nanohybrid composite restoration in molars after the placement of flowable compomer and composite using the snowplow technique.

Materials and methods: Twenty-four freshly extracted molars were taken and sectioned at the level of 2.5 mm from the coronal cusp and root tip. These sections were embedded in methacrylate and, after etching and bonding, were randomly divided into two groups (n=12) for placement of restorative materials. In group A, the plastic tube was filled with uncured flowable composite resin followed by the placement of packable nanohybrid composite in oblique increments. In group B, the plastic tube was filled with uncured flowable compomer followed by the placement of packable nanohybrid composite in oblique increments. Each specimen was then tested under a universal testing machine to determine the shear bond strength.

Results: Independent sample 't' test revealed a statistically significant difference between the mean shear bond strength of compomer and composite (P value<0.001), where flowable composite showed higher values compared to compomer.

Conclusion: It was seen that the use of composite showed a significantly better bond strength of the restoration when compared to compomer using the snowplow technique. Both materials and application techniques can influence the bond strength of a material as determined in this study.

## Introduction

Entering into the new era, a rise in the use of resin-filled materials has led to the utmost success. The very beginning of the restorative dentistry period marked the stabilization and retention of restoration which most often required the sound tooth structure preparations thereby providing large areas of undercuts. This problem is greatly resolved after the introduction of newer materials which bind micromechanically to the tooth structure. The attainment of a perfect bond on the tooth and the restoration is the primary goal of restorative dentistry for the prevention of dislodgement of the material from the tooth interface [1]. Also, due to increasing awareness and patient's demands for more esthetic restoration and good strength, an increased use of materials, such as composites and compomers, has raised [2]. Composite resins own better mechanical properties and better esthetics than many other types of cement, but they need bonding agents as they are hydrophobic and hence fail to adhere to the teeth [3]. As there is the involvement of several clinical steps for good bond procurement, compomers were introduced in 1992 [4]. Compomers are polyacid-modified resin composites. The physical properties of compomer and composite resin are similar as they are thought to bond to dentin by micromechanical bonding. As resin composites are used in this, the polymerized acid monomer shows acidity when it comes in contact with the saliva and there is a reaction of fluoride-containing basic glass resulting in the cariostatic effect [5]. When a normal tooth is subjected to masticatory forces, it transfers the occlusal biting load through the enamel and then into dentin which further gets distributed over a large internal volume of tooth structure as compression resulting in lowering the effect of local stresses. In contrary to this, when a restored tooth is exposed to such forces, it tends to transfer the forces along with the tooth-restoration interface, therefore leading to complex stress distribution in the form of shear stress, compression or tension [6]. Many attempts have been made to decrease the bond strength discrepancy as well as porosity and microleakage related to resin restorations. This involves several techniques for reducing polymerization and decreasing the C-factor, following the incremental placement technique that leads to the reduction of some residual stress at the interface between the tooth and the restoration. Among these techniques, a newer technique also known as the snowplow technique has been introduced. The “snowplow technique” is placing flowable composite in a layer, on the gingival margin of the proximal box as well as on the pulpal floor of a composite resin restoration. This layer of the composite is uncured before the placement of a composite restorative material that is denser filled [7]. Hence, the entire motive of this study is the determination and comparison of shear bond strength between two restorative materials, that is, composite resin and compomer, by using this new kind of technique.

## Materials and methods

**Figure 1:**
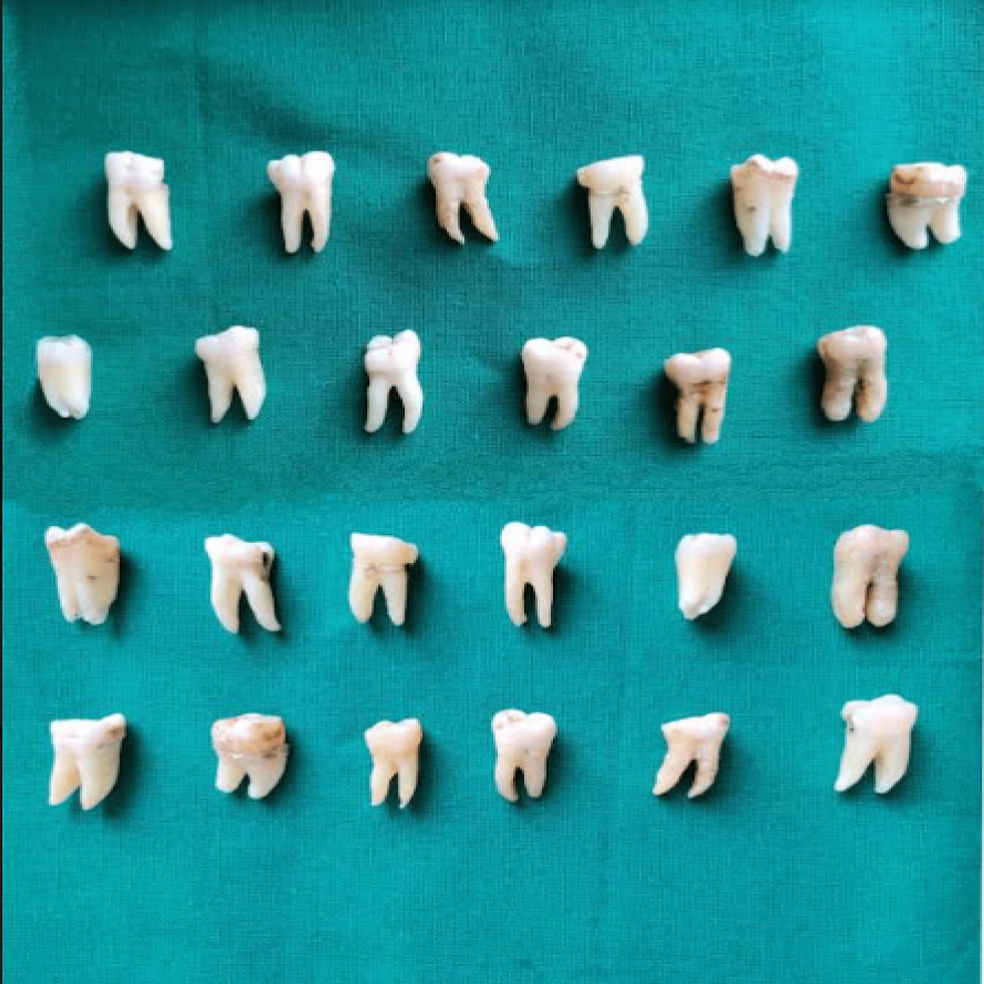
Samples were collected and cleaned

The present study was conducted in the Department of Conservative Dentistry and Endodontics at Sharad Pawar Dental College and Hospital. The ethical approval to perform the study was obtained from the Datta Meghe Institute of Medical Sciences Ethical Committee (DMIMS(DU)/IEC/2021/132). Twenty-four freshly extracted molars were taken (Figure 1). The teeth that were caries free and unrestored were chosen for the study. The teeth with restorations, cracks, enamel and dentin fractures were excluded from the study. The teeth were cleaned with the help of scaler tips to clear all tissue tags and debris and then stored in 0.9% normal saline.

Specimen preparation

**Figure 2:**
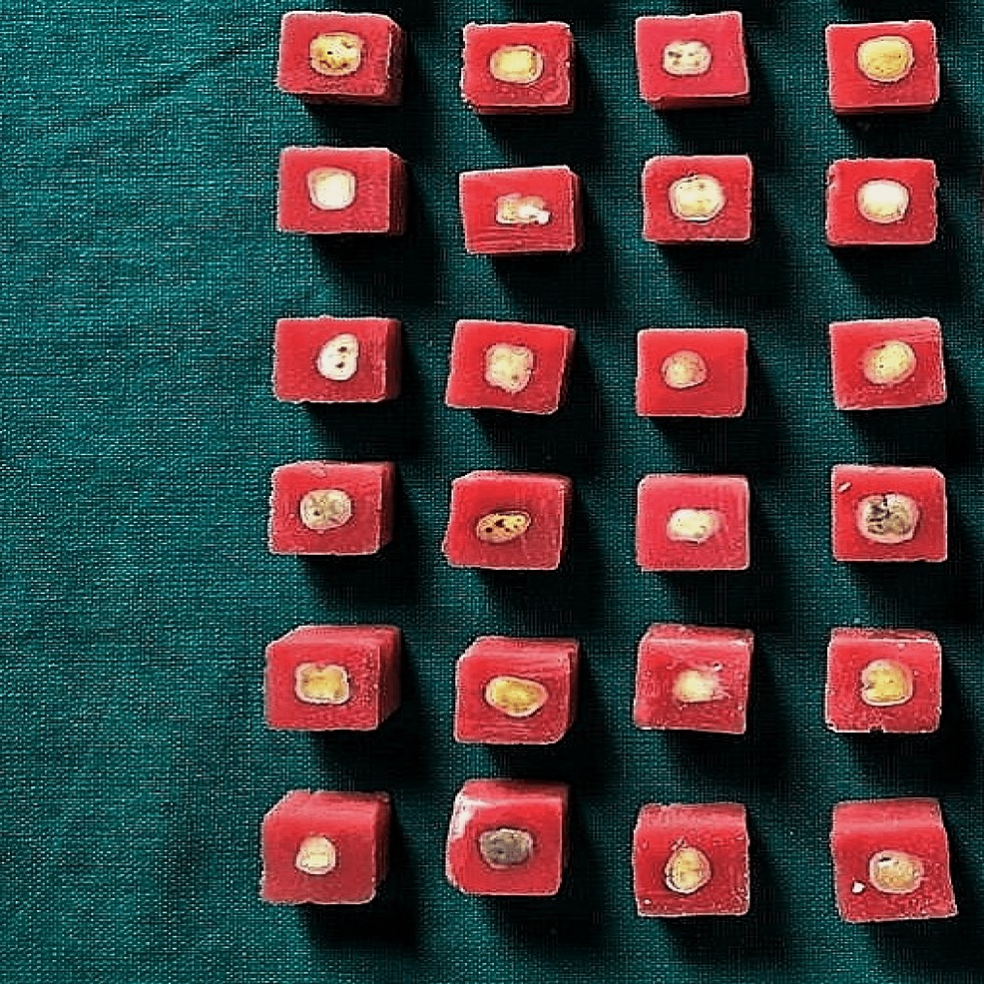
Tooth sections embedded in methacrylate-based molds

The sectioning of the tooth was done using a diamond disc (Mani, Japan) of 0.2 mm thickness along with water coolant. Each sample was sectioned at the level of 2.5 mm from the coronal cusp and root tip in order to attain a uniform surface area in all the samples and a disc of 2 mm width was formed. After applying a cold mold seal (Pyrax, India) as separating medium to steel mold of size 17 mm × 20 mm × 20 mm, the sectioned tooth was kept inside the mold and cold cure powder and liquid were poured. Molds were placed in water to cool, when methacrylate had set. After 20 minutes, samples were carefully removed from the mold die. The grinding of samples was then done to expose maximum dentinal surface followed by finishing with silicon carbide emery paper of 320, 600 and 1200 grit. Once tooth-embedded methacrylate base was formed, they were separately labeled for the composite and the compomer groups and thereafter gently dried before the placement of the restorative materials. No separate cavity was made on the surface of the prepared tooth and methacrylate base (Figure 2).

Bonding procedure

Etching was done for 15 seconds using 37% phosphoric acid (Total Etch, Ivoclar Vivadent, Liechtenstein) and then the surfaces were rinsed with water for 10-20 seconds. After this, the prepared surfaces were dried again. A bonding agent (3M ESPE, Saint Paul, USA) was then applied and cured with a light curing device for 20 seconds. A plastic tube of 3 mm × 2 mm (internal diameter × height) was placed on each sample, and all the samples were randomly divided into two groups: Group A: The plastic tube was filled with flowable composite resin (3M ESPE Filtek Z350, Saint Paul, USA) and left uncured (snowplow technique). This was followed by the placement of packable nanohybrid composite (3M ESPE Filtek Z250 Xt, Saint Paul, USA) in oblique increments and then curing each layer for 20 seconds. Group B: The plastic tube was filled with flowable compomer (Dyract Flow, Dentsply Sirona) and left uncured (snowplow technique). This was followed by the placement of packable nanohybrid composite (3M ESPE Filtek Z250 Xt, Saint Paul, USA) in oblique increments and then curing each layer for 20 seconds. After storing each sample in distilled water for a time span of 24 hours, it was thermocycled with 1,500 thermocycles in the temperature range of 12°C ± 2 - 60°C ± 2 with 30 seconds immersion and at an interval of 10 seconds in between baths.

Shear bond testing

**Figure 3:**
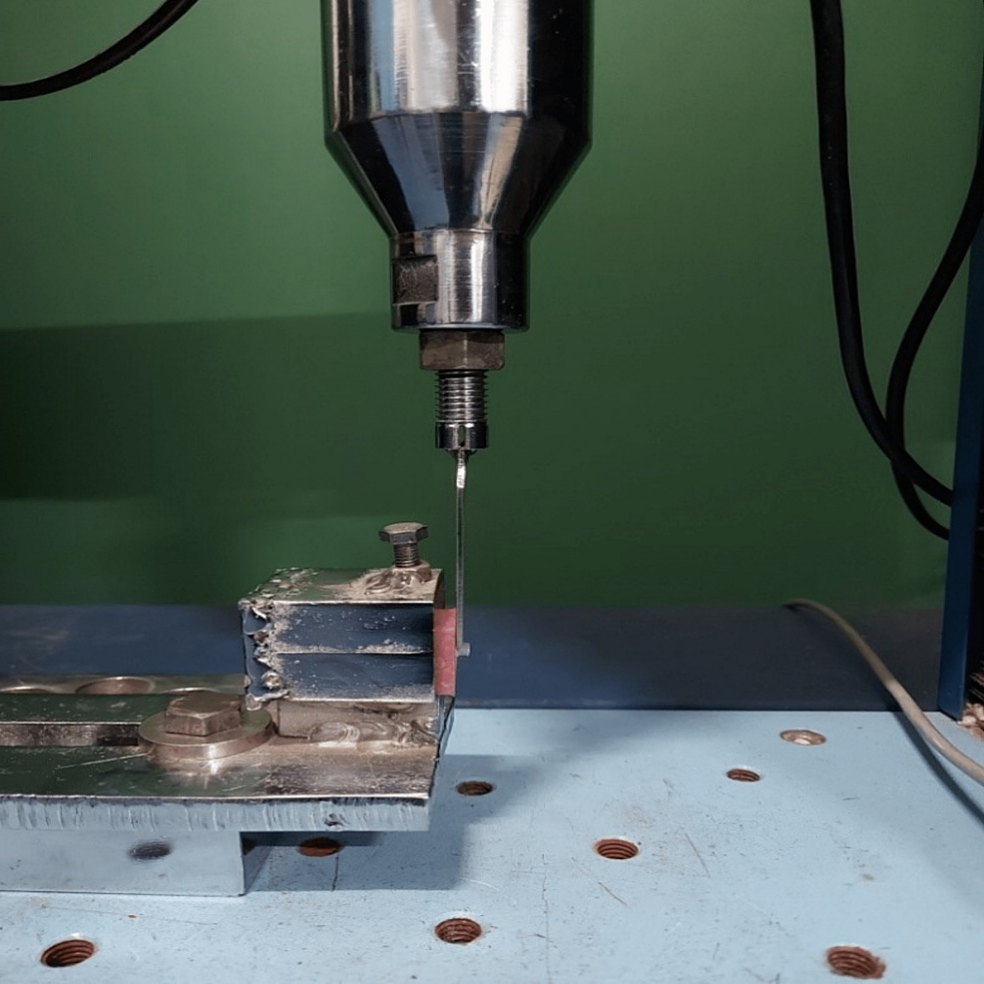
Universal testing machine

The shear bond strength test for every specimen was carried out using a universal testing machine (model no. UNITEST-10, Korea), with a cross-sectional area of 12.56 mm^2^. The dimensions of all the specimens were entered into the program for processing and computation. The two supporting wedges had a distance of 20 mm in between them, and the crosshead speed was preset at 1 mm/min. A chisel-shaped rod was aligned in the crosshead so that the force delivered to the specimen was immediately parallel and adjacent to the dentin surface as well as perpendicular to the long axis of the tooth until failure occurred. The specimens were connected to the load measuring cell, which continuously recorded the load applied to the specimens. Shear bond strength was calculated using the following formula: shear bond strength=load (N)/surface area (mm^2^) (Figure 3). The shear bond strength was recorded in megapascals (MPa). The maximum load that was given to the samples was 218.75 N in both groups. Two sets of six different loads were used for each specimen in both groups as this helped in the evaluation of shear bond strength even in the presence of different loads rather than one constant load like in any other study. To evaluate the shear bond strength between the two groups, a statistical analysis of the data was done and students unpaired 't' test was conducted. The values of standard deviation and mean were calculated.

## Results

The independent sample 't' test revealed a statistically significant difference between mean shear bond strength values of compomer and composite (P value<0.001). Here, the bond strength values revealed that the composite had slightly higher mean shear bond strength (13.57 ± 0.89 MPa) compared to the compomer (10.22 ± 0.65 MPa). The overall highest occurring shear bond strength recorded in this study was 14.96 MPa for composite. The mean shear bond strength with the dentin of the two respective materials is hereby presented in Table 1.

**Table 1:** Master chart for shear bond strength (MPa) of groups A and B N: Newton; MPa: megapascal.

Sample ID	Maximum Load (N)	Shear Bond Strength (MPa)	Sample ID	Maximum Load (N)	Shear Bond Strength (MPa)
No.1	80.80	14.32 MPa	No.1	80.80	10.7 MPa
No.2	186.25	13.38 MPa	No.2	186.25	9.8 MPa
No.3	218.75	14.96 MPa	No.3	218.75	10.8 MPa
No.4	129.65	14.09 MPa	No.4	129.65	10.14 MPa
No.5	125.60	13.02 MPa	No.5	125.60	9.56 MPa
No.6	135.55	13.9 MPa	No.6	135.55	9.8 MPa
No.7	80.80	14.6 MPa	No.7	80.80	10.01 MPa
No.8	186.25	12.16 MPa	No.8	186.25	11.06 MPa
No.9	218.75	13.45 MPa	No.9	218.75	11.55 MPa
No.10	129.65	13.98 MPa	No.10	129.65	9.43 MPa
No.11	125.60	12.9 MPa	No.11	125.60	9.77 MPa
No.12	135.55	12.16 MPa	No.12	135.55	10.05 MPa

The descriptive values of the shear bond strength of the two groups are depicted in Table 2.

**Table 2:** Descriptive values of shear bond strength for the two groups MPa: megapascal.

	Group A (Composite) Shear Bond Strength	Group B (Compomer) Shear Bond Strength
Mean	13.57	10.22
Standard deviation	0.89	0.65
Standard error	0.25	0.18
Minimum	12.16 MPa	9.43 MPa
Maximum	14.96 MPa	11.55 MPa

The comparison of shear bond strength between the two groups using students unpaired 't' test is depicted in Table 3.

**Table 3:** Comparison of shear bond strength between composite and compomer using student t test

Parameters	Composite	Compomer	Difference	P value
Shear bond strength	13.57 ± 0.89	10.22 ± 0.65	3.35 ± 1.10	<0.001

## Discussion

Ever since restorative dentistry has evolved, there has been a constant and unending forage for a material that will truly adhere to the tooth structure. The principle behind good adhesion is a close approximation at the interface of the material with the walls of the cavity and hence resulting in a minimum tooth-restoration gap interface [8]. This provides a better bond between them and accounts for a minimum amount of leakage from that area and increased shear bond strength. Composite is the most popularly used restorative material. Hence, the current study was performed to analyze the influence of bond strength on composite restorations compared to compomer restorations.

Shear bond strength is important to the restorative material clinically because of the fact that the major dislodging forces at the tooth restoration interface have a shearing effect. Therefore, higher shear bond strength implies better bonding of the material to the tooth [9]. In the present study, the samples selected were molars to obtain a greater surface area of dentin. The samples were subjected to thermocycling that helps to mimic hydrolytic degradation occurring within the resin-based materials, especially in oral environment. Also, thermocycling gives a better degree of conversion of composite resin [10]. After this, the samples were subjected to shear bond strength testing. Sufficient bond strength is needed for the resin to withstand the internal stresses exerted due to polymerization shrinkage. Also, shear stress is regarded to simulate much of the clinical circumstances and is comparatively easy to carry out [11].

Shear bond strength cannot be detected inside the oral cavity or cannot be measured in vivo. Thus, bond strength detection tests are performed outside the oral cavity and are beneficial for evaluating the outcome of adhesive systems. The recommendation by Rueggeberg [12] was followed in this study. It is suggested to perform the testing of bond strength after tooth sectioning either 1 mm below the dentinoenamel junction or 1 mm above pulp horns to allow homogeneity for the following testing. In a shear bond test, two materials are connected via an adhesive agent and loaded in shear until a fracture occurs by occlusal chipping. It was the most widely used test over other tests as no further specimen processing was required after the bonding procedure [13].

The two materials, composites and compomers, were used in this study pertaining to the earlier hypothesis that different properties of materials such as composition, elastic modulus, and setting reaction can influence their bond strength [14]. In our study, the shear bond strength of compomers was found to be in the range of 9-12 MPa. This result was in accordance with Triana et al., where the compomer’s shear bond strength to dentin varies between 11 and 21 MPa [15]. In contrary to this, Frankenberger et al. investigated the shear bond strength of compomers with dentin and found it to be higher in the range of 29-31 MPa [16]. Also, the shear bond strength of composite with dentin in this study was in the range of 12-15 MPa. This result was in accordance with Schneider et al., who found that composite has significantly higher values than compomer on comparing the shear bond strength of compomer and composite [17]. Also, similar results were seen when Prabhakar et al. compared the shear bond strength among composite, compomer and resin-modified glass ionomer cement and found that composite had the highest bonding strength when compared to the other two [18]. In addition, it is hypothesized that minimum bond strength of 15-20 MPa is required for both dentin and enamel to minimize the shrinkage pressure of composite resins [19]. Therefore, in accordance with the investigations mentioned above, compomers show similar or lower shear bond strength than composites.

The quantity of methacrylate that may undergo photoactivated polymerization varies between different materials. As a result, not only does the pretreatment of the dentin affect bond strength but so does the resin composition of the components [20]. Furthermore, conditioners containing monomers, such as hydroxyethylmethacrylate (HEMA), appear to be significant in the creation of hybrid layers that can penetrate demineralized dentin and/or produce a micromechanical bond in compomer materials with adhesive systems of high shear bond strength. The composites treated with phosphoric acid, on the other hand, have more firmly adhered material on the dentin surface than the composites treated with polyacrylic acid. This could be due to the composite material's deeper penetration and micromechanical interlocking when treating the dentinal surface with phosphoric acid [21].

Also, an oblique incremental technique was used for the final restoration of the tooth with nanohybrid packable composite. It is widely accepted that incremental composite resin filling decreases shrinkage stress as a result of reduced polymerization material volume. Each increment layer is compensated by the next, and the consequence of polymerization shrinkage is comparatively less damaging as then only the volume reduction of the last layer can damage the bond surface. This technique reduces the C-factor and prevents the distortion of cavity walls [22].

Co-curing a flowable liner and overlying the composite resin together, the snowplow technique showed a better result comparatively. Due to the increased viscosity of the overlying composite, the uncured liner penetrates better into the dentinal tubules and promotes sealing at the borders. The gap between the tooth-restoration interface would be less if the flowable liner was cured independently, resulting in increased bond strength [23,24]. Since no research has been done earlier on the shear bond strength of flowable composites and compomers using the snowplow technique, this study has helped us to evaluate and draw a conclusion as to which material has a better shear bond strength comparatively.

Clinical significance

Since posterior teeth are mostly subjected to a shearing phenomenon during mastication, placement of the restorative material with better shear bond strength is recommended for these teeth to minimize and elude restoration failure as well as increase patient satisfaction. The difference of 3 MPa between compomer and composite in the current study is definitely considered clinically significant. However, the in vitro results cannot be directly applied to clinical situations, and hence, a comprehensive evaluation of the restorations should be conducted to conclude their performance.

Limitations

Limitations of the present study include the limited sample size. Since the configuration factor also plays a major role in polymerization shrinkage, further research is needed to determine the influence of the configuration factor on bond strength testing. As there are various composition and properties of different resin composites and compomers, further investigations with different materials using the same snowplow technique are also required.

## Conclusions

High bond strength is essential for the successful outcome of different restorations. According to this study, the bond strength of compomer was seen to be less than that of composite comparatively. The snowplow technique also had an influence in determining the shear bond strength of both compomer and composite. Therefore, it is seen that different materials and their placement techniques can both influence the bond strength of a material.
